# Financing strategies to improve essential public health equalization and its effects in China

**DOI:** 10.1186/s12939-016-0482-x

**Published:** 2016-12-01

**Authors:** Li Yang, Li Sun, Liankui Wen, Huyang Zhang, Chenyang Li, Kara Hanson, Hai Fang

**Affiliations:** 1 School of Public Health, Peking University Health Science Center, Beijing, China; 2 London School of Hygiene and Tropical Medicine, London, UK; 3 China Center for Health Development Studies, Peking University, No 38 Xueyuan Road, Haidian District, Beijing, China

**Keywords:** Financing strategies, Equalization, Public health services, Effects, China

## Abstract

**Background:**

In 2009, China launched a health reform to promote the equalization of national essential public health services package (NEPHSP). The present study aimed to describe the financing strategies and mechanisms to improve access to public health for all, identify the strengths and weaknesses of the different approaches, and showed evidence on equity improvement among different regions.

**Methods:**

We reviewed the relevant literatures and identified 208 articles after screening and quality assessment and conducted six key informants’ interviews. Secondary data on national and local government health expenditures, NEPHSP coverage and health indicators in 2003–2014 were collected, descriptive and equity analyses were used.

**Results:**

Before 2009, the government subsidy to primary care institutions (PCIs) were mainly used for basic construction and a small part of personnel expenses. Since 2009, the new funds for NEPHSP have significantly expanded service coverage and population coverage. These funds have been allocated by central, provincial, municipal and county governments at different proportions in China’s tax distribution system. Due to the fiscal transfer payment, the Central Government allocated more subsides to less-developed western regions and all the funds were managed in a specific account. Several types of payment methods have been adopted including capitation, pay for performance (P4P), pay for service items, global budget and public health voucher, to address issues from both the supply and demand sides. The equalization of NEPHSP did well through the establishment of health records, systematic care of children and maternal women, etc. Our data showed that the gap between the eastern, central and western regions narrowed. However the coverage for migrants was still low and performance was needed improving in effectiveness of managing patients with chronic diseases.

**Conclusions:**

The delivery of essential public health services was highly influenced by public fiscal policy, and the implementation of health reform since 2009 has led the public health development towards the right direction. However China still needs to increase the fiscal investments to expand service coverage as well as promote the quality of public health services and equality among regions. Independent scientific monitoring and evaluation are also needed.

## Background

Over the past 65 years, the public health system in China has made significant progress to enhance health for the entire population. After the founding of the People’s Republic of China in 1949, the Chinese government made various innovations for better delivery of public health services. For example, at the beginning of 1960s, China launched a village doctor training program to create a front-line workforce, providing public health services and essential medical services including clinical treatment and drugs [1, 2]. In addition, disease prevention and primary care were the two most important tools at that time and people were able to receive some basic vaccines to prevent infectious diseases. All of these interventions lead to great health outcomes in China [3].

However, the public health system was ignored due to the transition from the planning economy to the market economy in the 1980s and 1990s. The government funds in the public health sector declined, which led public health institutions to generate their own revenues (i.e. selling vaccines, providing more profitable services) [4]. Some infectious diseases such as Tuberculosis (TB), re-emerged as a result of poverty and health inequities [5–7]. Fortunately, the Chinese government eventually realized that issues in the health care system must be addressed (particularly public health) and made various corrections.

After the 2003 Severe Acute Respiratory Syndrome (SARS) pandemic, the Chinese government paid more attention to public health and allocated more funds to public health sectors. In the 2009 health care reform policy, an essential public health package, including nine types of basic services and six types of catastrophic services, was launched. The PCIs including community health care centers, township hospitals and village clinics provided basic services and the specialized public health institutions like centers for disease control (CDCs) provided catastrophic services. The government regulated the guideline for basic services and provided training for public health workers. The financial supports were shared by the central and the local governments. Until 2015, the package included 12 types of basic services and seven types of catastrophic services. The budget per capita for basic services increased from 15 Renminbi (RMB) in 2009 to 40 RMB in 2015. Almost every Chinese citizen has equal access to this essential public health package. By summarizing China’s experiences and lessons learned during development of both public health service systems and financing strategies, especially with regard to improving universal access, the present study will provide significant policy implications for public health development and health systems strengthening in other developing countries.

Health equity analysis was often used to assess the improvement of healthcare or public health equalization, which is concerned with four focal variables: health outcome, health care utilization, subsidies received through the use of services and payments people make for health care [8–10]. The equity analysis methods include Lorenz curves and Gini coefficients, Thiel index, the index of dissimilarity(ID), the slope indices of inequality(SII), relative index of inequality(RII) and concentration index(CI) [8]. Since the policy has been implemented for only 6 years, the process indicators instead of health outcomes will be mainly considered for effects measurement. Because of data availability, we just measure the financing equity of essential public health services and summarize the experiences and lessons by using mixed methods.

## Methods

### Conceptual framework

**Fig. 1:**
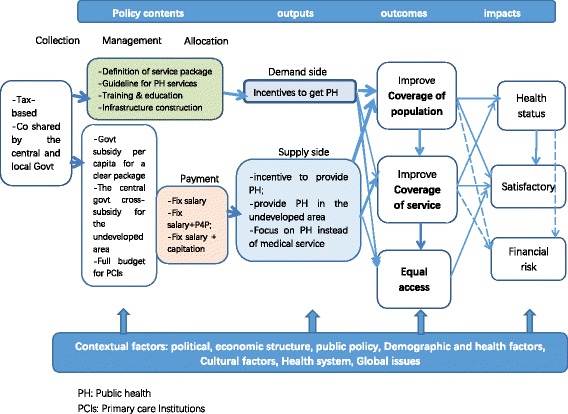
Conceptual framework

Based on the theory of change, we formed a theoretical framework of public health financing. Policy contents, including financing strategies for fund collection, management, and allocation, which could provide incentives for both the supply side and demand side and finally influence the outcomes and impacts. Contextual factors will indirectly contribute to outcomes by affecting the policy contents (Fig. 1).

### Review

The review included studies concerning China’ public health equalization in either Chinese or English on databases of PubMed, Medline, China National Knowledge Infrastructure(CNKI), and Wan-fang data. In addition, the review is confined to studies concerning financing strategies which improve access to public health and health outcome from 1959 to 2015 in China. The keywords are:” public health equalization” or “public health” or “primary healthcare”, and “revenue collect”, or”fund collect” or “revenue manage” or”fund manage” or “revenue allocate” or “fund allocate” or “financing mechanism” or “health finance”, and “population coverage” or “coverage rate” or “service content” or “service package” or “service items” or “access” or “availability” or “cost sharing” or “out of pocket” or “financial risk protection” or “catastrophic spending”. Policy articles or other documents and reports on public health revenue collection, management, allocation, or financing strategies for improving access to public health for all were included. Two reviewers identified titles and abstracts of all articles from the search, and retrieved the full text articles. Finally, we obtained a total of 208 literatures studies after data screening. The following literature information has been collected from relevant studies including background, content, mechanism and effect of the policy interventions. The main results and conclusions in the reviewed studies have been extracted. We used mixed-method syntheses to summarize successful financing strategies to improve access to public health for all in the past 65 years especially since 2009 NEPHSP policy in China [11, 12].

### Interview

We interviewed six experts in the public health field with semi-structured questionnaire, including two officials from China National Health and Family Planning Commission, two experts from national health account department at China National Health Development Research Center, one director from China community health association and one director from expand preventive immunization(EPI) department in China CDC. 1.5–2 h were spent for each interview. The questions for interview include:

1. How long has you worked there? What was your duty at that department?
2. Why did China implement the public health equalization policy?
3. What are the changes in public health?
4. How was fund collected, managed and allocated?
5. What were the provide side and the demand side’s responsiveness on this policy?
6. What are experiences or lessons for the policy implementation, which aspects still need improvement?

We recorded it, coded it and conducted qualitative content analyses.

### Secondary data analysis

We collected data from China Health Statistics Yearbook, 60 Years of New China Yearbook, National Health Service Survey report, National Health Financial Report, National Health Account Report and Global Burden of Disease (GBD) database by Institute for Health Metrics and Evaluation (IHME) at Washington University in St. Louis, United States. In addition, we searched secondary data on some Non-Governmental Organizations (NGO) and government websites [13]. By collecting data from above statistic reports and websites, we could show evidences on equalization process for essential public health financing and health indicators improvement since 2009. We used Gini coefficients through the slab method to assess the total financing equity for public health in China [8, 14]. and calculated the Thiel index to assess the financing equity among different regions [8].

## Results

The results include three parts: 1) reviewing the three phrases of public health financing evolution from 1949 to 2015, 2) summarizing the experiences and lessons of financing strategies learned during development of Essential Public Health Equalization and 3) assessing effects on government public health expenditure, expanded services coverage and narrowed the gap of health indicators between the urban and rural area. We generated the first part mainly by literature review, the second part based on literature review and key informants interview, and the third part based on literature review and second data analysis.

Equal access to basic services is one principle in the public health system of China. One of core policies is the free provision of basic public health services to all residents. With the development of the policy over the past 6 years, China has achieved almost universal basic public health services coverage for its population of 13.73 billion with increased funding levels, expanded services, and enhanced financial equity. The experience from China can provide policy lessons for other developing countries.

### Foundation for basic public health services: sustainable public funds

As part of public health, public health financing should be responsibilities of various levels’ governments. Lacks of sustainable financing for public health will affect the access and equity of public health service. China has some lessons as well as experience in the past 65 years. From 1949 to the present, China’s public health financing has undergone three phases.

#### Planned economy period after the founding of the People’s Republic of China (1949–1984)

The central government collected funds to address major public health issues and launch the “Patriotic Health Campaign”, which effectively decreased mortality from infectious diseases and significantly improve health status for the entire population. The life expectancy at birth of the Chinese people has been extended from 35 years in 1949, to 67 years in 1980, The World Bank and the World Health Organization called it the “China Model”, characterizing this strategy as maximizing health benefits with limited costs, which could be applied across many developing countries [15–17].

#### Financial system reform and market liberalization period (1985 to 2002)

After national government budget reforms favoring decentralization and tax redistribution, Chinese local governments failed to take full responsibility for funding the public health system. The government contribution to total public health expenditures decreased sharply. This weakened the role of PCIs for the provision of public health services. In addition, the emphasis of public health institutions shifted to clinical treatment instead of prevention. Without consistent financial supports from central budgets, the PCIs were incentivized to become self-financing entities. Because of the stagnation or even decline of basic public health service provision, some infectious diseases such as TB re-emerged [4, 18, 19].

#### Reinforcement period after SARS (2003 to present)

Based on an idea of the “Harmonious Society”, and people-centered political and social policies, the government plays more active roles in the public health system and attaches great importance to this sector again. Expenditures for public health institutions and PCIs are again funded by the national budget. In addition, the government has increased the overall investments in public health, enhanced the primary health care system, trained health workers, and promoted health development in rural areas [20, 21].

### Financing strategies for equalization of essential public health services

Equalization of essential public health services means every Chinese citizen, regardless of their gender, age, race, occupation, place of residence, and income level, can receive the same essential public health services, as mandated and supported by the government. In view of the differences in people’s needs for public health services, vulnerable groups such as low income people are given more attention [22]. Essential public health services are mainly provided by PCIs including urban community health service centers (stations), township hospitals and village clinics free of charge [23].

The current public health system in China includes a network of 3492 disease surveillance centers, 1271 professional public health institutions (such as tuberculosis dispensaries), 27,215 hospitals and 912,074 primary care facilities [24]. In specialized public health institutions, government budgets fully cover staff salaries, construction and capital development, pooled general funds, and major public health campaigns such as control of Acquired Immune Deficiency Syndrome (AIDS), TB and endemic diseases. Public hospitals undertake particularly required public health services that are publicly subsidized. As for PCIs, the government allocates funds for human resources as well as construction and capital development by government budget. The government allocates operating funds by government purchasing service.

Before 2009, the construction funds for PCIs were mainly from subsidies of the central government, and the operational costs and personnel expenses were mainly from local governments’ usual appropriation and medical services revenue generated by PCIs themselves. The usual fiscal appropriation was not enough to pay for personnel expenses. In Sichuan Province, for example, the annual fund in rural areas was only 0.5 RMB per capita [25]. The PCIs lost money due to high services costs and these losses seriously affected their initiatives to provide more public health services [26].

**Fig. 2:**
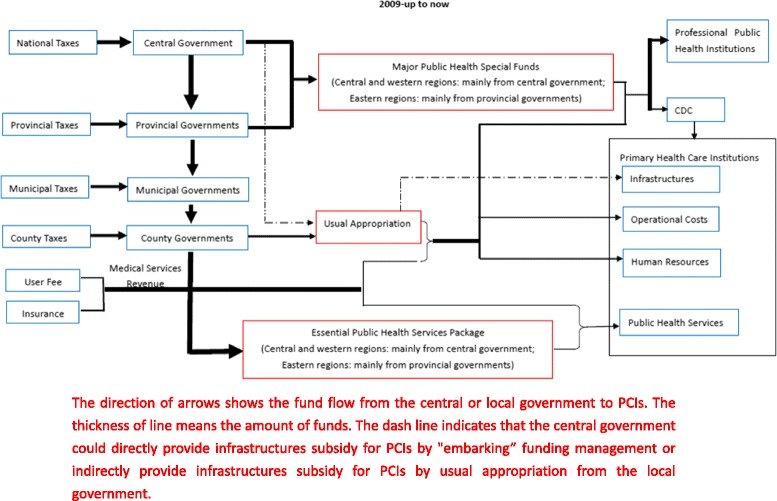
Essential public health financing since 2009

In 2009, the new special funds for NEPHSP were added into the public health sector. The funds are managed by special transfer payments through China Ministry of Finance. Cross uses between funds are not allowed any more by "earmarked” funding management system from top to bottom. The national, provincial, municipal and county governments allocate the funding to local fiscal sectors directly according to a per capita fund standard based on the total number of the resident population [23] and the local fiscal sectors pay the PCIs for providing public health services based on mixed payment of fix salary, pay for performance(P4P) and capitation (Fig. 2).

Details of the financing strategies for basic public health services in fund collection, management and allocation are discussed below.

#### Clarification of services included in the basic public health package

In 2009, China launched the NEPHSP with nine items, including health records establishment, health education, immunization, child health, maternal health, geriatric health, hypertension and type 2 diabetes management, severe mental illness management, and the surveillance and control of infectious diseases and public health emergencies. The service package has been continually expanded. In 2012, health supervision and management was added. In 2015, a regulation of traditional Chinese medicine and TB management was added into the public health service package, which currently included a total of 12 items (Table 1) [27–29]. By service comparison we can see that not only the service items but also the coverage of essential public health services was expanded from 2009 to 2015. For example, the target services group for children's systematic care extended from 0–3 years to 0–6 years.

**Table 1:** Essential public health services

Before 2009	2009	2011	2015
Establishing health records for all citizens	Establishing health records for all citizens	Establishing health records for all citizens	Establishing health records for all citizens
Health education	Health education	Health education	Health education
Children's health management	Children’s health management (0–36 months)	Children's health management (0–6 years old)	Children's health management (0–6 years old)
Maternal health management	Maternal health management	Maternal health management	Maternal health management
Vaccination	Vaccination	Vaccination	Vaccination
Reporting and handling of infectious diseases	Reporting and handling of infectious diseases	Reporting and handling of infectious diseases and public health emergencies	Reporting and handling of infectious diseases and public health emergencies
	Health Management for elderly people	Health Management for elderly people	Health Management for elderly people
	Health management for patients with hypertension	Health management for patients with hypertension	Health management for patients with hypertension
	Health management for patients with Type 2 diabetes	Health management for patients with Type 2 diabetes	Health management for patients with Type 2 diabetes
	Management for patients with severe mental illness	Management for patients with severe mental illness	Management for patients with severe mental illness
		Health Supervision and Management	Health Supervision and Management
			Traditional Chinese medicine management
			TB management

National clarification about the minimum service coverage has promoted the targeted provision of public health services and facilitated the process of assessment. In addition, local governments can add other public health services into this basic national package according to their local financial capacity and public health conditions.

#### Establishment of minimum funding level with progressive gradual increases

A national funding level was set by a standardized cost formula of each service item. The minimum funding level was 15 RMB (approximately 2.38 USD, 1 USD =6.3 RMB in 2015) per capita in 2009 and has increased to 40 RMB (6.35 USD) per capita in 2015 [23, 30]. The central government requires that every locality meets this minimum level, in order to guarantee implementation. Province and municipality level governments can further supplement the funding level according to the content of their local basic public health service packages, cost of services and local financial capacity, which has helped to expand services in the package for many areas. For example, a study suggested that the cost of the package in Beijing was 50 RMB (7.95 USD) per capita in 2010 based on survey in 17 sample centers and model estimation [31].

#### Shared responsibility and transfer payment

National, provincial, municipal and county governments in China share responsibility for funding basic public health services, and the national government allocates more money to less-developed middle and western regions by transfer payments. The proportions contributed by governments at different levels vary among regions, partially based on local socio-economic status. Funds allocated from the central government via general or special transfer payments account for 80% of total basic public health expenditures in western regions, 60% in central regions, and only10–50% in the more prosperous eastern regions. This helps to alleviate funding disparities and gaps in western and central regions [32] (Table 2). Similarly, the provincial governments can cross-subsidize counties by transferring funds from richer to poorer areas by transfer payments. Taking the 2009 minimum public health funding level of 15 RMB per capita as an example, contributions to western regions from the national, provincial and local levels of government were 12 RMB, 2 RMB and 1 RMB respectively. By comparison, only 9 RMB was from the national government in central regions. In eastern areas, the majority of the 15 RMB minimum came from local governments [32] (Table 3).

**Table 2:** Funding criteria for 2009 national essential public health services at all levels of governments in different regions (RMB)

	Western regions	Central regions	Eastern regions
Central finance	12 yuan per capital	9 yuan per capital	Six provinces (Fujian, Shandong, Liaoning, Jiangsu, Zhejiang and Guangdong) and three municipalities (Beijing, Tianjin, and Shanghai) are subsided by the central government according to their financial situation
Provincial finance	2 yuan per capital	6 yuan per capital	
Municipal and County finance	1 yuan per capital

**Table 3:** The funding proportions at different levels of governments in Hebei Province (2011 and 2012) (RMB)

	County directly under the management of provincial governments			County non-directly under the management of provincial governments		
	Poor financial situations	Relatively poor financial situations	Relatively good financial situations	Poor financial situations	Relatively poor financial situations	Relatively good financial situations
Provincial Finance	4.5	4	3.5	4	3.5	3
Municipal Finance	3	3	3	3.5	3.5	3.5
County Finance	2.5	3	3.5	2.5	3	3.5

#### Earmarked payments and strict allocation by capitation

Public health funds in China are managed as ‘special financial funds’, which means they are managed as ring-fenced budgets with unified accounting and strict allocation by capitation. This strong transparency in allocations can effectively reduce issues of payment delay or fund misappropriation. Moreover, it can help improve direct supervision of public financial departments, ensuring that disbursements are not impeded and flow smoothly and securely in the health system.

There are mainly two ways in the disbursement of funds for essential public health services. The first is that central and provincial project funds are directly appropriated by the provincial finance departments to municipal and county finance departments. The county finance departments allocated funds to PCIs in accordance with the results of the performance evaluation. The second is the establishment of municipal finance centralized payment accounts. Municipal finance departments directly allocated funds to PCIs. Take Tianjin city as an example, municipal and district governments match funds that are then turned in to the municipal finance centralized payment accounts and allocated directly to community health service centers. Municipal finance department keep accounts alone and do not adjust the use of funds. Municipal and district health boards take the responsibility of supervision [33]. This can ensure funding allocation in place and in time.

#### Pre-payment by capitation with subsequent top up

In order to avoid problems from the delay of disbursements and ensure the effectiveness of funding for basic public health services. A large proportion (50%) of public health funds are allocated by capitation at the beginning of each fiscal year. According to the performance assessment system, subsequent funds are linked to the facility’s actual delivery of services, which includes organization and management, responsible use of funds, productivity in completed tasks, quality, timeliness, socio-economic benefits, sustainable impact, social satisfaction, and other metrics. These payments can therefore increase the incentives to provide basic public health services in primary health care facilities and ensure funds are spent as intended by policymakers.

#### Government procurement of services and public-private partnerships (PPP)

The special fund for essential public health services were allocated by government procurement. Government procurement of public health services refers to the following two ways, government proposes specific tasks, objectives, requirements and assessment criteria, and PCIs provide free essential public health services to people. The government allocated the public health fund in terms of seven kinds of financial payment methods [34]: capitation, line budget, salary, pay for performance [33, 35], global budget [36, 37], fee for Service [38, 39] and public health voucher [14, 40, 41]. Actually mixed payment methods were often used in practice.

The government also purchase the public health services by signing a contract with the private sector such as village doctors and the latter receive a modest subsidy for providing public health services associated with the package. The willingness of village doctors to provide public health services has been improved since the introduction of the package and a minimum subsidy, although village doctors do not find the subsidy to be sufficient remuneration for their efforts [42–44].

Government procurement of services and public-private partnerships (PPP) can improve incentives in the private sector and alleviate shortages of health workers in public facilities. Before the current policy of essential public health service equalization, public funds were only available for staff salaries but not institutional management. As a result, strategic performance of the public health services suffered. After adoption of the policy, pooled government procurement of services has led to greater purchasing efficiency for public health services. Health workers in PCIs are additionally more motivated, because their compensations are linked to performance assessment. Furthermore, the government can purchase services provided by private sector actors such as village doctors, in order to effectively alleviate public health workforce shortages.

### Effects

The evaluation system of NEPHSP policy can effectively evaluate, interpret and improve basic public health services. Hu Shanlian initially established the evaluation indicators for this policy by consulting with experts, relying on the conceptual framework of the health system financing [45]. Yu Yong combined the evaluation indicators with "national essential public health service standards" (2011 edition) to effectively evaluated current policies [46].

Both process indicators and outcome indicators are used to evaluate NEPHSP policy. Process indicators are mostly service utilization indicators, used to measure the process effects of resources allocation. Outcome indicators are used to reflect the final outcomes of the resource allocation. Since only 6 years for this policy implementation, process indicators are often used in current empirical studies [47–52].

#### The improvement of government public health expenditure equity

**Fig. 3:**
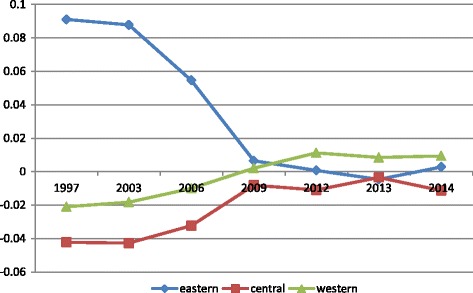
The Thiel index of GHE between eastern, central and western area in 1997–2014

Based on the 2014 price level, since 2003 the government health expenditure (GHE) and the government public health expenditure (GPHE) increased year by year and have increased more rapidly after 2009. The GHE increased from 146.8 billion RMB in 2003 to 611.2 billion RMB in 2014. The GHE per capita increased from 113.6 RMB (2003) to 446.9 RMB (2014) [53]. Measured by the Gini coefficient, we found that inequality in GHE fell from 0.33 (2003) to 0.10 (2014), and inequality in GPHE fell from 0.25 (2008) to 0.23 (2014). Measured by the Theil index, the gap of GHE between eastern, central and western areas has narrowed sharply since 2009 (Fig. 3).

#### The expanded coverage of essential public health services

**Fig. 4:**
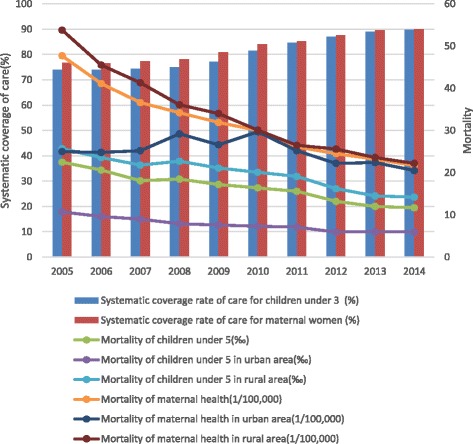
Systematic coverage rates of public health care for children under 3 and maternal women and mortality for children under 5 and maternal women

In 2015, adoption of standard electronic health records has reached to more than 75%. Systematic coverage rates of public health care for children under 3 years old and maternal women are above 85% (Fig. 4). The coverage rate for people over 65 years old remains at 65% while the immunisation rate among school-age children is above 90%. Standard management of hypertension and diabetes has reached 86.27 million and 24.19 million patients respectively, in an equivalent to management rates of 35% and 30%. Meanwhile, the standard management rate of registered patients with severe mental disorders has reached to 73% and 40% of patients covered by traditional Chinese medicine health care. Nine million TB patients, or 90% of total TB patients in China, are successfully managed. The hospitalized delivery rate among rural pregnant women has reached to 99% [54].

#### The narrowed gap of health outcomes between urban and rural area

As to outcome indicators for systematic care for children under 3 and maternal women, the mortality for children under 5 and maternal women decreased sharply in 2005–2014, especially in rural area, after 2009. The gaps between urban and rural areas have significantly narrowed since 2009, as shown in Fig. 4.

**Fig. 5:**
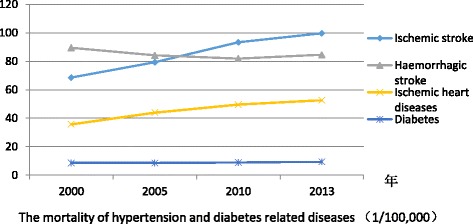
The change of mortality of hypertension and diabetes related diseases in 2000–2013

As to outcome indicators for systematic care of patients with hypertension and diabetes, the mortality of ischemic stroke and ischemic heart diseases increased in 2000–2013, except the mortality of haemorrhagic stroke has decreased since 2005, and mortality of diabetes increased slightly since 2005 (Fig. 5). As we know, the hypertension is the leading risk factor of haemorrhagic stroke (RR = 2.74) [52]. Total Cholesterol (RR = 2.7) and Triglycerides(Male: RR = 2.5, female: RR = 3.8) are more contributed to ischemic stroke compared with blood pressure (RR = 1.92) [55, 56]. Considering the control of dyslipidaemia is not included in the NEPHSP, it’s easy to understand that the mortality of haemorrhagic stroke decreased slightly and the mortality of ischemic stroke still increased. The gaps for mortality of hypertension and diabetes related diseases between urban and rural areas still existed in 2000–2013.

### Challenges

This public funding is nevertheless not enough in PCIs. Current workforce shortages and weakness in capacity will affect the quantity and quality of services that can be offered [42, 57, 58]. In addition, local governments may lack the capacity to effectively assess performance in terms of productivity and/or quality. Service coverage and financing mechanisms for China’s migrant population (approximately 252 million in 2015) also need to be improved.

## Discussion

Although many studies proved that the causal association between the public health expenditure and infant or child mortality [9, 10], some studies well summarized China’s experience on public health in 1949–1984 [3, 16] and lessons in 1985–2002 [4, 17], some studies assessed the effects of NEPHSP on service coverage and equity [14, 40–48], very few studies described China's financing strategies and mechanisms for the NEPHSP [34, 35, 41, 43]. This study could be an important contribution to the exiting literature on evaluation of public health equalization in China.

China’s experience of different financing strategies for public health shows that the public health sector can develop stably and sustainably only if the responsibility of government – especially at the national level – for financing is emphasised. In fact, the 2009 policy of basic public health services equalization was not a novelty, but rather the re-establishment of public financing responsibility and governance in China, in order to set a mechanism for equity in financial and service provision. Developing countries that rely on the national budget and/or international aid to mobilise resources for health expenditures can learn from China’s experiences [5, 6, 16]. However, it is worth noting that public health financing in China is influenced strongly by its unique national governance and public financial management.

### Implication for other low or middle income countries

#### Strengthening the government’s leading role in public health financing

The Chinese national government has introduced a clear and basic service package and clarified the service content, standards, and minimum financing levels, which has led to better health sector accountability [59]. The national government plays the main role in public health financing, and local governments should continue to be clear about their financing responsibilities. Financial equity across citizens and regions can be guaranteed by transfer payments facilitated by national or provincial governments [34, 35]. The national government sets policies for subsidy management, allocates central funds, and implements the management hierarchy across levels.

#### Integrated payment management to ensure full and timely funding is in place

Earmarked funding and allocation by capitation can increase transparency of funding levels, which can safeguard against the delay or diversion of funds [35]. Top up disbursement for actual services according to recurrent expenditure management can improve incentives in PCIs [37]. With this combination of pre-appropriation and later payments based on performance assessment, the process of disbursements can be accelerated to meet operational needs. Moreover, government procurement of services can promote PPP, to improve incentives for private sector actors to provide public health services as a supplement to public institutions [36, 38, 39].

#### Customised design of the basic public health service package

According to local conditions, in terms of funding criteria as well as implementation schedule and goals, it is essential to continuously improve the health system [36]. In a large country with significant regional diversity, the key point is to increase local governments’ incentives to promote equity of basic public health services [37].

### Limitations

It has been only 6 years since the carry out of NEPHSP equalization policy in 2009, it is difficult to use the data to measure the improvement of health outcomes and health equity in the public health sector. We need to use longitudinal data to capture its effectiveness in future. However based on existing evidences we could find that many process indicators has improved since 2009 which may finally result in improvement of health outcomes based on many experimental studies [50, 51, 56].

## Conclusion

Financing strategies are essential parts in the public health equalization policy. Public fiscal policies have a major effect on the delivery of essential public health service. In many middle or low income countries, people couldn’t acquire or have equal access to basic public health services due to the lack of sustainable public financing, which result in major infectious diseases and endemic diseases spreading, high maternal mortality and mortality of children, finally preventing the realization of MDG. The Chinese public health financing evolution proved that equalization of health outcomes depends on fiscal equalization, health financing equalization and equal access to public health services. And Chinese experiences for NEPHSP could provide lessons for other developing countries.

## Abbreviations

AIDS: Acquired Immune Deficiency Syndrome
CDCs: Centers for disease control
GBD: Global Burden of Disease study
GHE: Government health expenditure
IHME: Institute for Health Metrics and Evaluation
NEPHSP: National essential public health services package
NGO: Non-Governmental Organizations
P4P: Pay for performance
PCIs: Primary health care institutions
PPP: Public-private partnerships
RMB: Renminbi
RR: Risk ratio
SARS: Severe Acute Respiratory Syndrome
TB: Tuberculosis
